# Nasopharyngeal Pneumococcal Carriage among Healthy Children in Cyprus Post Widespread Simultaneous Implementation of PCV10 and PCV13 Vaccines

**DOI:** 10.1371/journal.pone.0163269

**Published:** 2016-10-05

**Authors:** Adamos Hadjipanayis, Elisavet Efstathiou, Maria Alexandrou, Loukia Panayiotou, Chrystalla Zachariadou, Panayiotis Petrou, Vasiliki Papaevangelou

**Affiliations:** 1 Paediatric Department, Larnaca General Hospital, Larnaca, Cyprus; 2 European University Medical School, 6, Diogenis Street, Engomi, 1516 Nicosia, Cyprus; 3 Microbiology Laboratory, Larnaca General Hospital, Larnaca, Cyprus; 4 Third Department of Paediatrics, National and Kapodistrian University of Athens, General University Hospital “ATTIKON”, Athens, Greece; Universidade de Lisboa Faculdade de Medicina, PORTUGAL

## Abstract

The objective of the study was to describe the incidence of pneumococcal nasopharyngeal carriage, serotype distribution and antibiotic resistance profile of pneumococcal nasopharyngeal isolates in healthy children aged 6 to 36 months following the implementation of conjugate vaccines. A nasopharyngeal swab was collected from 1105 healthy children following a stratified random sampling between September 2013 and April 2014. Demographics, vaccination status and data on possible risk factors were recorded. Isolates were serotyped and tested for antibiotic susceptibility. The nasopharyngeal carriage rate was 25.3%. Among 1105 children enrolled, 393 had received PCV13 and 685 PCV10. The prevailing isolated serotypes were: 23A (14.3%), 15A (8.9%), 6C (8.6%), 23B (7.5%), 19A (5.4%) and 15B (5%). The proportion of non-vaccine serotypes, PCV10 serotypes, PCV13 additional serotypes (3, 6A, 19A) was 76.8%, 2.1% and 10.4% respectively. Although children, who were fully or partially vaccinated with PCV13, were 63% less likely to be colonized with additional PCV13 serotypes compared to those vaccinated with PCV10, the difference is not significant (95%Cl = 0.14–1.02, p = 0.053). The highest antibiotic non-susceptible rates were found for erythromycin (28.2%) and penicillin (27.9%). The overall multidrug resistance rate was 13.2%, with serotypes 24F (4/6), 15A (14/25) and 19A (6/15) being the main contributors. Carriage rate was similar between children vaccinated with PCV10 or PCV13. The high incidence of 15A serotype which is also multidrug resistant should be underlined. Ongoing surveillance is needed to monitor the dynamics on nasopharyngeal carriage.

## Introduction

*Streptococcus pneumoniae* (Sp) is a common cause of childhood bacteraemia, meningitis, otitis media, pneumonia and sinusitis [1]. Young children and elderly people are at highest risk. It is estimated that 14.5 million cases of invasive pneumococcal disease (IPD) occur annually in children aged <5 years, resulting in approximately 500,000 deaths worldwide, mainly in developing countries [1].

The human nasopharynx is the main reservoir of pneumococcal carriage. Although nasopharyngeal (NP) carriage is asymptomatic, it represents a first and necessary step for the pathogenic process of pneumococci towards IPD [2]. There is a strong association between serotype distribution of pneumococci causing invasive diseases and those colonizing the nasopharynx of children [3, 4]. Moreover, it has been documented that the antibiotic resistance profile of pneumococci colonizing the nasopharynx reflects that of the bacteria grown in children with acute otitis media [5].

The highest incidence of pneumococcal colonization occurs in young toddlers. Consequently, this risk group is the most important source of horizontal spread of pneumococcal strains within the community [2].

In order to reduce the burden of pneumococcal disease, universal infant vaccination with a 7-valent pneumococcal conjugate vaccine (PCV7) was introduced in 2000 in the United States, resulting in a dramatic decline of IPD not only among vaccinated children but also in unvaccinated persons of all ages through herd immunity [6–9].

However, over the next years, cases due to non-PCV7 serotype strains (especially serotype 19A), increased [10, 11]. Of note, the overall decline in IPD cases, post PCV7 implementation, was higher compared to the small increase in IPD cases due to non vaccine serotypes (NVT) [8].

Subsequently, a 10-valent pneumococcal conjugate vaccine (PCV10) and a 13-valent pneumococcal conjugate vaccine (PCV13) replaced PCV7 in the routine infant immunization schedule offering wider coverage [12]. PCV10 contains three additional serotypes (1, 5, 7F) while PCV13 contains three additional serotypes to PCV10 (3, 6A, 19A).

Implementation of PCV universal vaccination has led to a significant decrease of IPD incidence and reduced NP carriage of vaccine serotypes (VT) among vaccinated children [13, 14]. However, there has been a slight or no change on the prevalence of pneumococcal carriage among asymptomatic carriers [15] since NVT incidence increased while the proportion of VT was substantially reduced [16–18].

In Cyprus, PCV7 was introduced in the national vaccine schedule in February 2004. It was recommended for universal vaccination of children aged 2–23 months and for children aged 24–59 months who are at increased risk for pneumococcal disease [19]. PCV10 was introduced in November 2009, while PCV13 in May 2010 and gradually replaced PCV7. The switch from PCV7 to PCV13 was completed following the recommendations of the Advisory Committee on Immunization Practices (ACIP)[20].

The PCV7 and thereafter the PCV10 were provided free of charge by the public health services. Initially, the 3+1 dose scheme was adopted and thereafter was modified to 2+1 dose. Both PCV13 and PCV10 were provided by private pediatricians who were vaccinating approximately 60% of children in Cyprus at that time.

Irrespective of the widespread implementation of pneumococcal vaccination, there are no published data regarding the incidence of pneumococcal NP carriage in Cypriot children. A nationwide population based study was conducted to describe the NP carriage rate, serotype distribution and the antibiotic resistance profile of NP carriage in healthy children aged 6 to 36 months, a decade after the introduction of conjugated pneumococcal vaccination.

## Materials and Methods

### Setting

Cyprus is an island, situated geographically at the Eastern Mediterranean Sea. It has an intense Mediterranean climate with hot, dry summers and mild rainy winters separated by short autumn and spring. The study was conducted to the areas of the Republic of Cyprus between the period September 2013 and April 2014. The population of the country is 865,900 inhabitants according to the last census (2012).

### Study design and population

Study population consisted of healthy children 6 to 36 months of age residing in the catchment area. Subjects were allocated in three age groups as follows: group A: 6–12 months, group B: 13–24 months, group C: 25–36 months.

Children were recruited from outpatient clinics of public hospitals, well baby clinics and paediatric private practices from all provinces of Cyprus. There are five public hospitals in Cyprus (one in each province). One paediatric outpatient clinic and one well baby clinic are available at each hospital and all of them accepted the invitation to participate in the study. Twenty-five out of 235 paediatric private practices were arbitrarily selected and accepted to participate in the study.

The sample size was estimated assuming that the carriage rate was 30%, a difference of 4% is detectable at 5% significance level with 80% power. In order to account for possible refusals 120 additional subjects were invited. A stratified random sampling was followed according to the population census corresponding to 5% of the population of each age group (stratum). Therefore, 269 children were enrolled in group A, 427 children in group B and 409 children in group C (out of 276, 433 and 416 invitations respectively). Children presenting with fever or any symptoms or signs of respiratory infection (acute otitis media, pneumonia, sinusitis), children who received antibiotics during the past 7 days (last antibiotic dose within 7 days prior to specimen collection) and immunocompromized children were excluded.

### Data collection

A field-tested questionnaire was completed by the child’s paediatrician, in order to collect information about demographic data, immunization status regarding Sp and possible risk factors for pneumococcal carriage. The immunization status of the children for Sp was recorded from the children’s health booklet. Potential risk factors examined included: day care attendance for subjects and their siblings, number of siblings, presence of a smoker in the house, history of breast feeding, respiratory infection diagnosed by a phycisian during the previous year (pneumonia, acute otitis media), antimicrobial use in the previous 3 months (7–30 days, 30–60 days, 60–90 days), history of hospitalization and the presence of health provider or teacher at home.

Childrens’ PCV vaccination status was determined as non-vaccinated, fully vaccinated and partially vaccinated. Children were considered partially vaccinated for age if they had not completed the schedule for age according to the Center of Disease and Control guidelines [20].

### Laboratory procedures

#### Specimen collection and bacterial identification

NP specimens were collected using a sterile rayon tip swab (Copan Ventury Transystem; Copan) on a flexible aluminium wire by trained pediatricians. Swab specimens were placed in transport medium (Sterilin Ltd UK, produced by Copan Italia S.P.A) and processed in the microbiological laboratory at Larnaca General Hospital within 24h. The swabs were inoculated into Columbia agar plates supplemented with 5% defibrinated sheep’s blood and incubated at 37°C in a 5–10% CO_2_-enriched atmosphere for 24–72h.

Suspected colonies of Sp were isolated in pure cultures, identification of which was based on phenotypic characteristics of the colonies having the following appearance: Small (0.5mm), round, grey—translucent colonies, moist or mucoid, surrounded by a green zone of alpha-hemolysis on the blood agar, usually presenting flattened or depressed center after 24h of incubation. The differential diagnosis from other species of *Streptococci* was confirmed by using the optochin (OPT) susceptibility test and solubility in bile (sodium deoxycholate) simultaneously. Optochin susceptibility test was performed by disk diffusion, using commercially available optochin discs (5 μg; 6 mm; Oxoid, Hampshire, England) applied onto blood agar plates incubated for 18–24 hours at 35–37°C with ~5% CO_2_. Isolates were considered to be resistant to optochin if they displayed inhibition zones smaller than 14 mm or larger than 14 mm with the presence of colonies inside the inhibition zone. For isolates with reduced susceptibility to optochin that appeared to be pneumococci, based on the other phenotypic observations prescribed, a bile (sodium deoxycholate) solubility test was performed. Bile soluble strains (Bile test positive) were reported as Sp. Strains partially soluble to the bile having optochin zones of inhibition of less than 14 mm were not considered pneumococci and were excluded from our study.

All the techniques prescribed above were tested with *S*. *pneumoniae* strain ATCC 49619 used as a positive control and *S*. *mitis* strain ATCC 49456 as a negative control.

Molecular techniques for identification and characterization of Sp were not used.

#### Antibiotic susceptibility tests

Antibiotic susceptibilities for Sp isolates were determined by the use of Kirby-Bauer disc diffusion method and by the automatic phoenix micro dilution system (BD Diagnostic System). All isolates were tested against oxacillin, penicillin, erythromycin, chloramphenicol, tetracycline, trimethoprim-sulfamethoxazole (TMP-SMZ), clindamycin and cefotaxime. Strains were screened for penicillin resistance using 1μg oxacillin discs. If oxacillin inhibition zones were <20mm, minimal inhibitory concentrations (MICs) of penicillin and cefotaxime were determined by the E test method (AB Biodisk, Sweden). MICs and breakpoints were interpreted according to the guidelines of Clinical Laboratory Standards Institute [21]. Briefly the non-meningitis criteria (oral use) for penicillin were: susceptible, MIC≤ 0, 06 μg/ml; intermediate 0.12–1 μg/ml, resistant ≥2 μg/ml and the non-meningitis criteria for cefotaxime were: susceptible, MIC≤ 1 μg/ml; intermediate MIC 2 μg/ml, resistant MIC ≥4 μg/ml. Non-meningitis criteria for oral use were adopted for penicillin. Isolates with MIC value higher that the susceptibility breakpoint were characterised as non-susceptible. Multidrug resistance (MDR) phenotype was defined as non-susceptibility to penicillin (MIC>0.12 μg/ml) plus resistance to ≥2 other non-β-lactam antimicrobial classes. Following the antibiotic susceptibility tests, one or two colonies of the isolated pneumococci were selected and frozen at -80°C for serotyping.

#### Serotyping

The isolates were initially grouped / serotyped by Latex agglutination using the pneumotest latex agglutination kit (SSI Diagnostica, Hillerod, Denmark) according to the published instructions [22]. The Pneumotest-Latex kit consists of 14 different pooled pneumococcus antisera (pools A to I and pools P to T) applied to latex particles. Thereafter, where multiple serotypes were observed, serotyping was processed by the capsular swelling method using serotype specific antisera (Statens Serum Institut, Copenhagen, Denmark). Due to budget restrictions some rare serogroups for this cohort were only tested by Latex-Kit (group 7, 9, 10, 12 and 33). All the pneumococcal isolates that did not react with the fourteen different pooled pneumococcus antisera were characterised as non-typeable.

Serotypes were grouped as PCV 10 serotypes (pneumococcal serotypes associated with PCV10), additional PCV13 serotypes (3,6A,19A), NVT and non-typeable. Isolates which were not serotyped were allocated according to their serogroups. A category “pool only” was created for those isolates only giving a pool sera result. Finally, isolates which did not survive through storage were categorized as data missing.

### Ethical approval

Ethical approval for the study was obtained from the Cyprus Bioethics Committee and permission to conduct the study was attained from the Ministry of Health. The participation in the study was voluntary and occurred after obtaining written informed consent by the parent(s) or guardians.

### Statistical analysis

All data relating to each individual participant were initially processed with the Epi-Info program and subsequently analysed using R software (version 3.2.2). Categorical variables are summarised as counts accompanied by percentages either marginally or jointly with other variables of interest. Continues variables are summarised with mean & standard deviation. Appropriate models / tests (e.g. chi-square or Fisher exact test or logistic regression) were employed to study differences of a response variable in various subgroups, allowing for multiplicity correction. All statistical tests were performed at the 5% level of significance.

## Results

### Study population and carriage rate

Out of 1,125 eligible children, 1,105 (98.2%) were recruited. Twenty parents declined study participation.

NP swabs were obtained for culture from all subjects. The mean age of children was 19.8 months (range 6–36 months). Distribution of enrolled children in the three predefined age groups and demographic data are shown in Table 1. Most children were residing in an urban area (64%).

**Table 1:** Pneumococcal NP carriage rate according to demographics and vaccination status.

Features	Total subjects	Carriers (%)
**Total population**	1105	280 (25.3)
**Gender**		
Male	589	160 (27.2)
Female	516	120 (23.3)
**Age group**		
Group A: 6–12 months	269	68 (25.3)
Group B: 13–24 months	427	91 (21.3)
Group C: 25–36 months	409	121(29.6)
**PCV uptake**		
Non vaccinated	23	11 (47.8)
Total vaccinated	1082	269 (24.8)
Total fully vaccinated	557	143 (25.7)
Total partially vaccinated	525	126 (24)
Total PCV 10	685	175 (25.5)
PCV 10 fully vaccinated	290	78 (26.9)
PCV 10 partially vaccinated	395	97 (24.6)
Total PCV 13	393	94 (23.9)
PCV 13 fully vaccinated	264	65 (24.6)
PCV 13 partially vaccinated	129	29 (22.5)

The overall carriage rate for Sp was 25.3%. Carriage rates in the various age groups are shown in Table 1. Of note, children 25–36 months of age were 1.55 times more likely to be carriers compared to children aged 13–24 months (95%CI 1.07–2.26, p-value = 0.017).

### Vaccination status

Only 23 children were unvaccinated with PCV, 557 children were fully vaccinated and 525 children were partially vaccinated (Table 1).

The carriage rate according to vaccination status is presented on Table 1. There is no statistically significant difference on overall NP carriage rate between any of the vaccinated groups.

### Possible risk factors

Incidence of all possible risk factors among children with and without Sp carriage is depicted in Table 2. Overall, 28.1% of children attended day care center (group A = 33/269, group B = 89/427, group C = 189/409) and 57.5% of children had ≥1 sibling. Day care center attendance, the presence of one or more sibling and the presence of a sibling attending day care center were identified as independent risk factors for pneumococcal carriage.

**Table 2:** Possible risk factors for Sp carriage.

Features	Numberof children	CarriersN (%)	OR	95% CI	p-value
**Siblings**					
0	469	88 (18.8)			
1	429	129 (30.1)	1,86	1.36–2.54	**0.01**
> = 2	207	63 (30.4)	1,89	1.30–2.76	**0.01**
**Day care attendance**					
No	794	150 (18.9)			
Yes	311	130 (41.8)	3,08	2.31–4.11	**0.01**
**Antibiotic consumption**					
None	507	121 (23.9)			
7–30 days	101	19 (18.8)	0,74	0.43–1.27	0.27
>31 days	497	140 (28.2)	1,25	0.94–1.66	0.12
**Passive smoke**					
No	557	141 (25.3)			
Yes	548	139 (25.4)	1,00	0.76–1.32	0.98
**History of breast feeding**					
No	345	92 (26.7)			
Yes	760	188 (24.7)	0,90	0.68–1.21	0.50
**History of pneumonia**					
No	1.083	272 (25.1)			
Yes	22	8 (36.4)	1,70	0.71–4.11	0.20
**History of acute otitis media**					
No	854	206 (24.1)			
Yes	251	74 (29.5)	1,32	0.96–1.80	0.09
**History of hospitalization**					
No	816	202 (24.8)			
Yes	289	78 (27.0)	1,12	0.83–1.52	0.45
**Presence of health provider at home**					
No	1.047	266 (25.4)			
Yes	58	14 (24.1)	0,93	0.50–1.73	0.83

### Serotype prevalence and distribution

**Fig 1:**
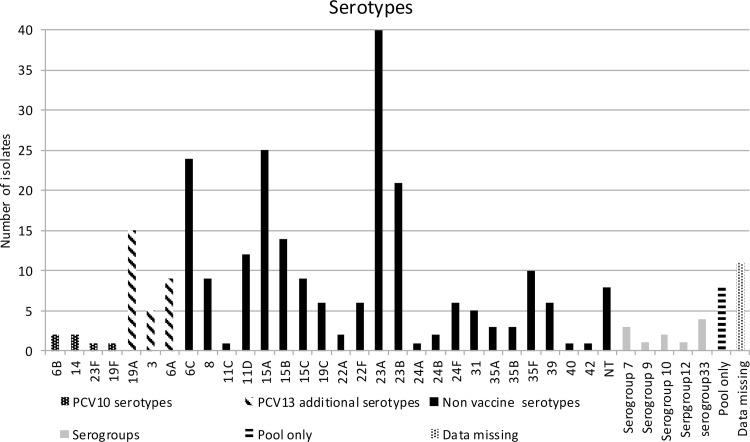
Number of different serotypes identified among Sp carriers of the cohort. PCV10 serotypes, PCV13 additional serotypes, serogroups, pool only and non vaccine serotypes (NVT) are presented with different shadow.

Serogrouping and serotyping was performed on 269 isolates. Eleven isolates did not survive through storage process and could not be serotyped (therefore categorized as serotype data missing). A total of 29 different serotypes were identified, 8 isolates were non-typable, 11 isolates were serogrouped and 8 gave only a pool sera result. Serotype distribution of all isolates is illustrated on **Fig 1.**

The six most predominant isolated serotypes, accounting for 49.7% of all isolates, were 23A (14.3%), 15A (8.9%), 6C (8.6%), 23B (7.5%), 19A (5.4%) and 15B (5%).

The proportion of NVT was high (76.8%). The overall carriage of PCV10 serotypes and PCV13 additional serotypes (3, 6A, 19A) was 2.1% and 10.4% respectively. The prevalence of PCV13 additional serotypes and 6C according to vaccination status is presented on Table 3. Among children fully vaccinated with PCV13, one isolate was identified as serotype 6A, two as serotype 19A and no isolate was identified as serotype 3, while in children fully vaccinated with PCV10 the numbers were 2, 5 and 2 respectively. The statistical comparison of prevalence for PCV13 additional serotypes and 6C among different vaccinated groups (PCV10 or PCV13, fully or partially) is also shown on Table 3. No statistical significance was found regarding the prevalence of 6C among children fully vaccinated with PCV10 in comparison to children fully vaccinated with PCV13. Moreover, no statistical significance was found regarding the prevalence of 19A between the above groups when we excluded children that were taking antibiotic at least 30 days prior to sampling.

**Table 3:** Comparison of prevalence for PCV13 additional serotypes and 6C among different vaccinated groups (PCV 10 vs PCV13).

Serotype	Fully vaccinated Number (%) PCV10 vs PCV13	Partially vaccinated Number (%) PCV10 vs PCV13	All vaccinated Number (%) PCV10 vs PCV13	OR			OR 95% CI			p-value		
				Full	Part	All	Full	Part	All	Full	Part	All
3,6A,19A	9 (11.5%) vs 3 (4.6%)	14 (14.4%) vs 2 (6.9%)	23 (13.1%) vs 5 (5.3%)	0.37	0.44	0.37	0.09–1.45	0.09–2.09	0.14–1.02	0.15	0.3	0.05
6C	5 (6.4%) vs 5 (7.7%)	13 (13.4%) vs 1 (3.4%)	18 (10.3%) vs 6 (6.4%)	1.22	0.23	0.59	0.33–4.45	0.03–1.88	0.23–1.56	0.76	0.17	0.3

### Antimicrobial susceptibility

Antimicrobial susceptibility testing was carried out for all isolates. Table 4 illustrates the detailed antibiotic susceptibility pattern of Sp isolates. The highest resistance rate was found for erythromycin (27.5%). There was only intermediate resistance to cefotaxime while no isolate resistant to chloramphenicol was found.

**Table 4:** Antimicrobial susceptibility of the 280 isolates.

Number (%) of isolates			
Agent	Sensitive	Intermediate	Resistant
Penicillin	202 (72.1)	69 (24.6)	9 (3.2)
Cefotaxime	270 (96.4)	10 (3.6)	0
Chloramphenicol	280 (100)	0	0
Tetracycline	239 (85.4)	5 (1.8)	36 (12.9)
Erythromycin	201 (71.8)	2 (0.7)	77 (27.5)
Clindamycin	222 (79.3)	3 (1.1)	55 (19.6)
TMP-SMZ*	212 (75.7)	20 (7.1)	48 (17.1)
Oxacillin	189 (67.5)	0	91 (32.5)

*TMP-SMZ: trimethoprim-sulfamethoxazole

Antimicrobial susceptibility of the 280 isolates among PCV10 serotypes, PCV13 additional serotypes, serogroups, pool only and NVT is described on Table 5. Out of 29 PCV13 additional serotypes, six were resistant and six intermediate to penicillin. All five serotype 3 isolates were susceptible to all antibiotics.

**Table 5:** Antimicrobial susceptibility of the 280 isolates among PCV10 serotypes, PCV13 additional serotypes, serogroups, pool only and non vaccines serotypes (NVT).

Serotype	N	penicillin		cefotaxime		cotrimoxazole		erythromycin		tetracycline		clindamycin	
		Intermediate	Resistant	Intermediate	Resistant	Intermediate	Resistant	Intermediate	Resistant	Intermediate	Resistant	Intermediate	Resistant
PCV 10	**6**	1	0	1	0	1	0	0	2	0	2	0	2
19A + 6A + 3	**29**	6	3	2	0	1	6	1	16	0	3	0	6
19A	**15**	3	6	2	0	0	6	1	8	0	3	0	6
6A	**9**	3	0	0	0	0	0	0	8	0	0	0	0
3	**5**	0	0	0	0	0	0	0	0	0	0	0	0
NVT	**215**	59	5	6	0	16	38	1	48	5	28	3	39
6C	**24**	5	0	0	0	2	3	0	3	0	0	0	1
8	**8**	0	1	1	0	0	2	0	0	1	1	0	3
11C	**1**	0	0	0	0	0	1	0	0	0	0	0	0
11D	**12**	0	0	0	0	0	0	0	1	0	0	0	0
15A	**25**	17	1	0	0	1	19	0	20	1	17	2	17
15B	**14**	4	0	0	0	1	2	0	1	0	1	0	1
15C	**9**	2	0	0	0	0	0	0	0	0	0	0	1
19C	**9**	0	1	1	0	1	0	0	2	0	2	0	1
22A	**2**	0	0	1	0	0	0	0	0	0	0	0	0
22F	**6**	0	0	0	0	1	0	0	1	0	0	0	0
23A	**40**	7	0	0	0	0	4	0	4	0	2	0	2
23B	**21**	11	1	0	0	6	4	0	3	0	0	0	2
24A	**1**	1	0	0	0	0	1	0	1	0	0	0	1
24B	**2**	0	0	0	0	0	0	0	0	0	0	0	0
24F	**6**	4	0	0	0	3	1	0	6	0	4	0	5
31	**5**	0	0	0	0	0	0	0	0	0	0	0	0
35A	**3**	2	0	0	0	0	0	0	0	0	0	0	0
35B	**3**	2	1	1	0	0	0	0	0	0	0	0	0
35F	**10**	0	0	0	0	0	0	0	0	0	0	1	0
39	**6**	1	0	1	0	0	1	1	3	3	0	0	4
40	**1**	0	0	0	0	0	0	0	0	0	0	0	0
42	**1**	0	0	0	0	0	0	0	0	0	0	0	0
Non-typeable	**8**	0	1	0	0	1	0	0	2	0	1	0	1
Serogroup 7	**3**	0	0	0	0	1	0	0	0	0	1	0	0
Serogroup 9	**1**	0	0	0	0	0	0	0	0	0	0	0	0
Serogroup 10	**2**	0	0	0	0	0	0	0	0	0	0	0	0
Serogroup 12	**1**	0	0	0	0	0	0	0	0	0	0	0	0
Serogroup 33	**4**	0	0	1	0	0	0	0	4	0	0	0	3
Pool only	**8**	0	1	0	0	0	0	0	1	0	0	0	0
Data missing	**11**	2	1	0	0	1	4	0	6	0	2	0	5
**Total**	**280**	**75**	**15**	**12**	**0**	**20**	**54**	**3**	**93**	**5**	**39**	**3**	**61**

The non-susceptible rates for NVT was high as shown in Table 5.

Serotype 23A, was the most frequent isolate found, with relatively low non-susceptible rates ranging from 5–17%. The second most frequent serotype was 15A which displayed a high resistant rate (72% to 80%).

The overall MDR rate was 13.2% (37/280). Major MDR serotypes were 24F (4/6, 66.7%), 15A (14/25, 56%) and 19A (6/15, 40%).

## Discussion

The overall pneumococcal carriage rate in our study was 25.3%. This is consistent with rates recently reported from other developed countries where universal vaccination of infants with PCV has been implemented [23, 24]. Moreover, in concordance with previous studies, the highest rate of pneumococcal NP colonization was observed in children 25–36 months old [25]. Children 25–36 month old had significantly higher rate of pneumococcal NP compared to 13–24 month old cohort (p-value<0.017).

Importantly, the herein presented carriage data includes children vaccinated with either PCV10 or PCV13. The overall NP carriage rate did not differ among children with different PCV vaccination coverage (fully or partially vaccinated, PCV10 or PCV13). This is in accordance with the majority of published studies evaluating the impact of universal immunization with PCVs. This may be due to the high incidence of NVT colonization in our cohort as described by other studies [16–18, 26].

To our knowledge, there is no published data regarding the immunization coverage for PCVs in Cyprus. In the present study, where a representative sample of children residing in Cyprus was enrolled, 98% of the cohort had received at least one PCV vaccine. Yet, only half of them were fully immunized according to age (Table 1). However, this estimation might be misleading since it considers “partially vaccinated” all children immunized with a 2+1 schedule. Although there is no official recommendation from the Immunization Committee of Cyprus Paediatric Society, many pediatricians have adopted this schedule. Moreover, children vaccinated in the public sector received the 2+1 schedule. So, if one accounts this group of children as fully vaccinated, the overall percentage of fully vaccinated children is 67.4%. Still, this is a vaccination coverage rate significantly lower than that reported from other European countries [27, 28].Therefore public health authorities need to stress the importance of timely vaccination coverage, which could enable the implementation of the 2+1 schedule. Since this is a cost saving schedule and in view of the current economic situation, a 2+1 schedule may be considered as vaccination coverage increases.

In accordance with previous data, in this cohort where most children had received at least one dose of PCV vaccine, attending day care or having a sibling attending remained a significant risk factor for NP carriage [29, 30]. In contrast with other studies recent antibiotic consumption and history of upper respiratory infection were not found to be associated with pneumococcal carriage [30, 31]. However, data on risk factors associated with NP carriage among unvaccinated and vaccinated children is controversial [29, 31].

In reference to serotypes identified, a high proportion (76.8%) of NVT was found among carriers as previously reported [17, 18, 30, 32]. Almost 50% of NP carriage was due to five serotypes, namely 23A/B, 6C and 15A/B (Fig 1) as described by others [17, 31]. Conversely to the above studies, serotypes 35B/F were less prevalent while we had no NP carriage by 11A. A risen incidence of cases of IPD caused by NVT, including serotypes 6C, 22F, and serogroups 15 and 33 has been described [26, 33–38]. Since there have been no studies on NP pneumococcal carriage in Cyprus prior to the introduction of PCV, no comments on possible serotype replacement can be made. However, as broadly discussed in the literature, the shift of NP carriage towards NVT stresses the need for ongoing awareness for changes in the epidemiology of invasive pneumococcal disease.

The high incidence of serotype 15A (8.9%) detected in our study is important since recent data have indicated that this serotype often colonizes vaccinated children and may cause multi-drug resistant Sp acute otitis media [39, 40].

In the current study, we had the opportunity to compare NP carriage among children vaccinated with the two available PCVs. Although children who were fully or partially vaccinated with PCV13, were 63% less likely to be colonized with additional PCV13 serotypes (3, 6A and 19A) compared to those fully or partially vaccinated with PCV10, the p value is not significant but this could be due to small sample size (95%Cl = 0.14–1.02, p value = 0.053). In addition no statistical significance was found comparing the prevelance of additional PCV13 serotypes among children PCV13 fully vaccinated with the children PCV10 fully vaccinated (95%Cl 0.09–1.45, p value = 0.15). Moreover, data from other areas where both extended PCV vaccines have been used, no significant difference in carriage rates of each serotype between groups of children that received PCV10 and PCV13 has been reported [32].

Furthermore, it has been shown that PCV13 prompts functional antibody activity against 6A/B/C and 19A/19F [41]. Of note in our population the prevalence of serotype 6C does not differ significantly among various vaccinated groups (PCV10 or PCV13, fully or partially). In addition a recent clinical study from France revealed a cross protection against serotype 6C as there was a decrease on prevalence of serotype 6C on NP carriage in children with acute otitis media who had received at least 1 dose of PCV13 [42]. Therefore more studies are necessary to shed light on the cross protection of PCV13 against serotype 6C.

Fifteen children carried serotype 19A, two of which were fully vaccinated with PCV13. A recent study, evaluating the impact of PCV13 on NP carriage revealed that serotype 19A was the only persistent VT in the study population [17]. Lee et al found that the implementation of PCV13 in children 6–23 months of age in Massachusetts between 2007 and 2011 resulted in increased colonization with non-PCV13 serotypes. However, serotype 19A remained the second most common serotype in 2011 despite the fact that vaccination coverage with PCV13 was high [18]. In contrast to the previous studies, an Italian study showed that serotype 19A was not detected in children fully vaccinated with PCV13 [30]. The persistence of serotype 19A despite the PCV13 vaccine is a critical issue given that it represents a known cause of invasive disease associated with all types of pneumococcal disease [43]. In the present study 13/15 children with 19A colonization were either unvaccinated (n = 1) or vaccinated by PCV10 (5 fully, 7 partially).

Αntibiotic resistance patterns of the Sp isolates cultured was studied. Serotype 19A is a highly resistant serotype to antibiotics. Six out of fifteen (40%) 19A serotypes were multidrug resistant strains while nine out of fifteen (60%) were either intermediate or resistant to penicillin. It is expected that the implementation of PCV13 will decrease the prevalence of antibiotic resistant Sp [44].

Of note, the resistance of NVT was due to the resistance of 15A serotype, which was the second most predominant among all isolates found in the study. Serotype 6C displayed relatively low non susceptible rates in our study in contrast to a recent study from Italy regarding the NP carriage after PCV13 implementation in which serotype 6C was the most frequent multidrug resistant isolate [30]. The possible replacement of carriage by non susceptible NVT is an issue of concern. In Portugal a decrease in the carriage of penicillin-resistant vaccine-type was observed following the introduction of PCV7 vaccine, but with an emergence of resistance in NVT [45]. However, Cohen et al reported a decrease in NP carriage and penicillin nonsusceptibility carriage among children with acute otitis media following PCV 13 immunization with dominant replacement serotypes 15A/B/C, 35B and 11A [42]. The above results could have also been influenced by the differences in antimicrobial consumption in the two countries. France implemented a massive campaign against inappropriate use of antibiotics and it was associated with a marked reduction of unnecessary antibiotic prescriptions, particularly in children [46]. On the other hand, Frazao et al reported a very high antimicrobial consumption in Portugal, emphasizing that reduction in the carriage of resistant pneumococcus may require a combination of the conjugate vaccine and a decrease of antibiotic consumption [45]. Unfortunately there is no published data for antimicrobial consumption in Cyprus.

The limitations of the study should be acknowledged. This is a baseline study examining the epidemiology of Sp carriage in the community. Since data prior to universal vaccination is unavailable, dynamics in NP carriage rates or serotype distribution could not be evaluated. Additionally, since both PCV10 and PCV13 are widely used in Cyprus, it is rather difficult to discern the effectiveness of the two vaccines on unvaccinated population or even vaccinees. Most importantly, these data are not coupled with National IPD data. Serotypes from serogroups 7 and 9 (n = 4) were not determined. This prevented the possibility of an analysis of which isolates from these serogroups were vaccine isolates. Finally, the laboratory methods used allowed only for detection of one predominant colonizing pneumococcal serotype and therefore colonization by multiple strains could not be identified.

The strength of this study is that this is the first pneumococcal surveillance study with representative samples per geographical region, according to the age-specific population distribution of the National Registry and therefore it is a starting point for future studies. Additionally, due to the concurrent utilization of PCV10 and PCV13 we had the opportunity to study NP carriage in two different vaccinated groups residing in the same area.

## Conclusions

The aforementioned data indicates that 10 years post PCV implementation in Cyprus, NP carriage rate in a representative sample of children was 25,3%. The coverage of PCV vaccination in Cyprus could be improved. Carriage rate did not differ whether children had been vaccinated with PCV10 or PCV13. An overall predominance of NVT was found in all vaccinated groups. The high incidence of 15A serotype which is MDR should be underlined.

The data derived from the present study provide a baseline comparison status for further evaluations regarding the effect of PCVs. Ongoing surveillance for pneumococcus NP carriage and invasive diseases is important in order to evaluate epidemiologic changes and to propose future vaccine strategies.

## Abbreviations

Sp: *streptococcus pneumoniae*
IPD: invasive pneumococcal disease
NP: nasopharyngeal
PCV7: 7-valent pneumococcal conjugate vaccine
NVT: non vaccine serotypes
PCV10: 10-valent pneumococcal conjugate vaccine
PCV13: 13-valent pneumococcal conjugate vaccine
PCVs: pneumococcal conjugate vaccines
VT: vaccine serotypes
